# Comparison of the Clinical Effectiveness of Intra-Articular Injection with Different Substances After TMJ Arthroscopy: A Systematic Review and Meta-Analysis

**DOI:** 10.1007/s12663-023-02047-7

**Published:** 2023-12-12

**Authors:** Juan Pablo López, María Paula Orjuela, Luis Vicente González, Alberto Jose Peraza-Labrador, David Díaz-Baez

**Affiliations:** 1 Hospital Universitario Fundación Santa Fe de Bogotá, Bogotá, Colombia; 2 Universidad El Bosque, Bogotá, Colombia; 3 Hospital Universitario La Samaritana, Bogotá, Colombia; 4 Department of Oral Research, School of Dentistry, Institución Universitaria de Colegios de Colombia UNICOC, Bogotá, Colombia; 5 Centro de Odontología Integral, Acarigua, Portuguesa Venezuela; 6 Department of Diagnosis Sciences, Texas A&M University College of Dentistry, Dallas, TX USA; 7 Unit of Basic Oral Investigation (UIBO) Facultad de odontología, Universidad El Bosque, Bogotá, Colombia

**Keywords:** TMJ arthroscopy, Hyaluronic acid, Sodium hyaluronate, Platelet-rich plasma, Plasma rich in growth factors

## Abstract

**Objective:**

This systematic review aims to describe the clinical outcomes after TMJ arthroscopy followed by intra articular infiltration with different substances.

**Materials and Methods:**

A literature search was carried out, the variables were Arthroscopy with different substances, pain and maximal mouth opening. The inclusion criteria were articles that reported infiltration of different substances after arthroscopy. Case series, observational studies, and randomized clinical trials were included. Exclusion criteria were studies that included arthrocentesis, animal studies, connective tissue disease, patients with previous surgeries.

**Results:**

Of the 5 studies finally included, the population studied were 346 subjects, of which 315 were female. The mean age was 34.7 (16–77). Regarding diagnoses, Wilkes III and Wilkes IV were taken into account. The most commonly used substance was sodium hyaluronate/hyaluronic acid in 4 of the 5 studies.

**Conclusion:**

Multiple substances have been infiltrated within the temporomandibular joint, with sodium hyaluronate/hyaluronic acid being the most studied. However, the benefit of substances like ATM artroscopia adyuvantes has not been clearly established. It is recommended in future studies that the substances and results be evaluated in the same way to obtain more homogeneous studies.

## Introduction

One of the most frequent TMJ disorders is internal derangement, which can cause a variety of clinical problems. In a systematic review and meta-analysis, degenerative joint disease has been related to disk displacement in approximately 66% of the cases. Also, imaging findings of degenerative articular diseases should be addressed early [1]. For the management of these entities, it is important to determine the most effective treatment. Recently, a meta-analysis of a network of clinical trials was published. That study advocates the use of minimally invasive therapies for the early management of degenerative disorders that include arthrocentesis and arthroscopy, with or without the application of intra-articular substances [2].

Concerning arthroscopy, Onishi introduced the technique in 1975 for direct vision of the articular structures to obtain an effective diagnosis [3]. Later, McCain et al*.* described the reposition of the articular disk by arthroscopy in a data collection with 11 patients, giving great diagnostic and operative versatility to this tool. Currently, a large number of authors have performed diskopexy with arthroscopy [4]. One of the techniques available was published in 2016 demonstrating that arthroscopy with resorbable pins is a useful procedure for improving clinical parameters and mandibular function with a decrease in pain and an increasing evolution in the mandibular interincisal opening in a short and long follow-up period [5, 6]. On the other hand, pain reduction, an increase in maximal mouth opening, and reductions in joint noise are some of the clinical variables improved with advanced arthroscopy [7].

Substances infiltrated in arthrocentesis have been extensively studied in the literature. However, the benefit of infiltrated substances exclusively in arthroscopy has not yet been studied to find out if they offer an additional benefit or if the benefit is due to arthroscopy. This systematic review and meta-analysis aim to describe the clinical outcomes exclusively after TMJ arthroscopy followed by intra-articular infiltration with different substances reported until now.

## Materials and Methods

This protocol has been registered in the PROSPERO database with ID CRD42021265201, following the PRISMA criteria under the following research question:

P: Adult patients with temporomandibular internal disorders with an arthroscopy indication.

I: Temporomandibular joint arthroscopy followed by intra-articular injections.

C: Operative or diagnostic arthroscopy with intra-articular infiltrations of different substances.

O: Impact on the clinical conditions of the patient is evaluated by variables such as pain and maximum mouth opening.

*Focused question*: Which of the substances usually injected intra-articularly after arthroscopy shows better clinical results, such as pain relief and maximal mouth opening (MMO)?

### Criteria for Selecting Studies

- *Inclusion criteria* The inclusion criteria were articles that reported infiltration of different substances only after arthroscopy procedures in patients over 15 years, describing visual analog scales (VAS) and (MMO), including clinical cases, case series, observational studies, and randomized clinical trials with at least 3 months of follow-up. The selection process began with articles published in the English language between January 1989 and December 2020.
- *Exclusion criteria* Studies that included arthrocentesis, animal studies, connective tissue disease, and articles that included patients with previous surgical treatment.
- *Types of substances* Hyaluronic acid (HA) and sodium hyaluronate (SH), platelet-rich plasma (PRP), plasma rich in growth factors (PRGF), corticoids, and analgesics

### Search Strategy

A systematic review was performed according to the PRISMA statement, and the protocol was enrolled and recorded in PROSPERO-CRD42021265201. A search of the Web of Science, MEDLINE, and EMBASE databases was done. The MEDLINE searches included a combination of relevant search terms. The search was completed on 21/09/2022. The results were limited to human-subject, and English-language articles. All abstracts were analyzed, and full-text articles were obtained when inclusion criteria were fulfilled. The references of the subsequent full-text articles were reviewed to identify additional relevant articles.

#### Search Methods for the Identification of Studies

A generic search strategy composed of controlled vocabulary exploded as Mesh (Medical Subject Headings) and free language, considering synonyms, abbreviations, acronyms, spelling, and plural variations, was designed. Individual search strategies were developed for each source of information (Appendix 1**).**

#### Critical Appraisal and Assessment of the Risk of Bias in the Included Studies

All studies were evaluated independently and duplicated by two reviewers (JPL and MPO) to determine methodological quality using the ROB2 tool (Version 2 of the Cochrane risk-of-bias tool for randomized trials) for randomized clinical trials. This tool is based on the evaluation and qualification of clinical studies, considering five domains, and focuses on assessing aspects that are relevant to the risk of bias in a study of this type (trial design, conduct, and reporting). For each domain, judgment can be ‘Low’ or ‘High’ risk of bias, or it can express ‘Some concerns’, and likewise, in each domain, there is a space for the evaluator to provide his or her personal opinion about it. Finally, any disagreements among the reviewers were subjected to the evaluation of a third evaluator (LVG).

##### Data Collection Process

The list with the bibliographic references identified in the electronic searches was downloaded into a library of the Rayyan^®^ program, where duplicate publications were eliminated, and an initial screening was carried out. In the first instance, the reviewers identified eligible articles by title. Afterward, each of the authors separately assessed the abstracts of these articles and selected potentially eligible studies. The reviewers subsequently independently verified the eligibility criteria (inclusion and exclusion) by reviewing each full-text publication (Table 1).

**Table 1:** Summary of demographic information of all studies included

Study	Study design	Subjects	Gender		Mean age	Arthroscoscopy sample	Diagnostic	Previous treatment
			Female	Male				
Miyamoto et al.[12]	Case series	63	57	6	26.0 years (16–50)	83 joints	DDwoR* Wilkes III	Non- surgical treatment for 3 months
Morey-Mas et al. [8]	Randomized controlled trial	40	37	3	35.3 years (SD, 13.3 years)	Treatment Group = 20 Control Group = 20	DDwR^a^ or DDwoR* Wilkes III or IV	Non-surgical treatment for 6 months (occlusal splint, medication, physiotherapy)
Fernandez-Sanroman et al. [9]	Randomized controlled trial	92	86	6	35.8 years. (17–67)	Group A = 42 Group B = 50	DDwoR* + OA^b^ WIlkes IV	Non improvement with NSAIDs physiotherapy occlusal splint for 6 months
Fernandez-Ferro et al. [10]	Randomized controlled trial	100	88	12	35.5 years (18–77)	PRGF = 50 HA = 50	DDwoR* or DDwR^a^ + OA^b^ WIlkes IV	Non improvement with NSAIDs, physiotherapy occlusal splint for 6 months
Castaño-Joaqui et al. [11]	Randomized controlled trial	51	47	4	41 years (18–76)	Control group = 25 Test group = 26	ID^c^ Wilkes III or IV	Conservative therapies for 3 months (soft diet, physiotherapy, occlusal splint, NSAIDs)
Total		346	315	25		366		

*disk Displacement without Reduction

^a^disk Displacement with Reduction

^b^Osteoarthritis

^c^Internal Derangements

##### Data Extraction

The characteristics of the selected evidence were summarized according to what was reported in the original publications using a standardized data extraction format in chronological order. The data collected included author, year, study design, population, type of substance used (*HA/SH, PRP, PRGF, corticoids, and analgesics)*, preoperative and postoperative VAS, preoperative and postoperative MMO, and follow-up period. In studies where the data corresponding to the summary and dispersion measures were not precisely specified, their graphical representations were used to extract the data using a plotdigitizer (https://plotdigitizer.com/app).

##### Data Synthesis

For the main comparisons between the results of the different combination techniques, see Table 2, accompanied by a narrative synthesis. Subsequently, two random effects meta-analyzes were performed to compare weighted means between the pre- and postoperative periods of the VAS and MMO variables using the RevMan 5.4.1 software developed by the Cochrane Collaboration. Each meta-analysis considered two subgroups, one to estimate the effect of PRGF and HA; the other groups were not considered due to the heterogeneity between presentations or the lack of data availability. A difference in measures (MD) with a confidence interval (CI) excluding 0 was considered statistically significant.

**Table 2:** Summary of outcomes

Study	Substance	Mean preoperative VAS	Mean postoperative VAS	Mean preoperative MMO	Mean postoperative MMO	Additional treatment	Observations	Follow-up
Miyamoto et al. [12]	400 ml of lactated Ringer’s solution + Hyaluronic acid (25 mg) 83 joints	Severe or moderate preoperative pain	90.5% = No pain 7.9% = Reduced pain 1.6% = Pain the same	27.2 ± 5.4	44.4 ± 4.1	Occlusal stabilization splint and physical therapy	Significant improvement in MIO (*p* < 0.001)	34.4 months
Fernandez-Sanroman et al. [9]	PRGF (5 ml) 42 joints Sodium chloride 5% (5 ml) 50 joints	PRGF = 7.7 ± 1.6 Control = 8.1 ± 1.9	PRGF = 1.2 ± 1.9 Control = 1.5 ± 2.3	PRGF 26.4 ± 6.3 Control = 27.2 ± 7.6	PRGF = 37.2 ± 3.9 Control = 36.1 ± 4.2	Capsulotomy, Miotomy, coblation	No significance differences between groups in MMO	2 years
Fernandez-Ferro et al. [10]	PRGF 50 joints (inferior space 1 ml and superior space 5 ml) HA 1% 50 joints (inferior space 1 ml and superior space 5 ml)	PRGF 8.14 ± 0.93 HA 8.35 ± 0.64	PRGF = 1.55 (± 1.9) HA = 2.20 (± 1.43)	PRGF = 27.74 ± 4.65 HA 27.92 ± 5.08	PRGF = 37.23 ± 4.94 HA = 36.54 ± 5.78	Capsulotomy, Miotomy, coblation	PRGF is more effective than HA in pain reduction	18 months
Morey-Mas et al. [8]	Sodium Hyaluronate (1 ml in the superior joint space) 20 joints Ringer’s lactate 20 joints	SH = 62.0 Control = 54.8	SH = 19.0 Control = 9.6	N/M	N/M	N/A	The reduction in joint pain was statistically significant in the SH group on the days 14 and 84	168 days
Castaño-Joaqui et al. [11]	Ringer’s lactate alone, 25 joints HA (20 mg/ml in the superior joint space) 26 joints	HA = 6.1 (SD 1.7) Control = 6.5 (SD 1.8) Overall = 6.3 (SD 1.7)	Overall = -4.08 (12 month visit)	Approximately 28 mm	Overall = 10.99 (12 month visit)	Pharmacological therapy, soft diet and home-exercises	No benefit of HA as an adjuvant therapy to arthroscopy during follow-up months 3–12	12 months

Abbreviations: *HA* Hyaluronic acid, *SH* Sodium Hyaluronate, *SD* standard desviation, *PRGF* Plasma rich Grow-factors, *MMO* Maximal mouth openning, *VAS* visual analog scale

## Results

### Description of the Selection Process

**Fig. 1:**
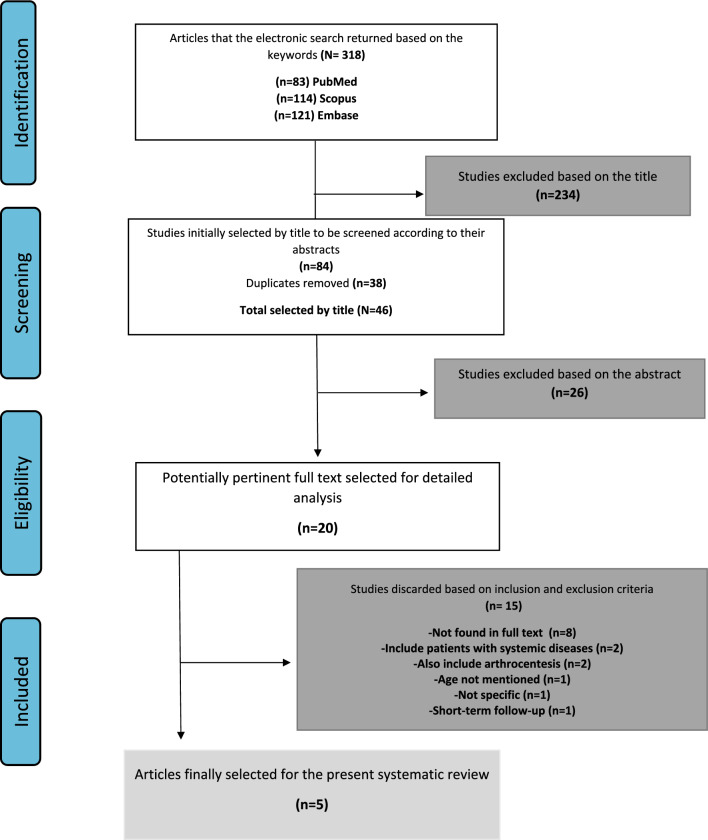
Flow chart diagram

The study selection process and resume are shown in a flow chart Fig. 1. A total of 26 studies were excluded based on the abstract, and 20 were potentially pertinent full texts selected for detailed analysis. Finally, only five articles were selected based on the inclusion and exclusion criteria.

### Description of Studies

Most of the studies were randomized clinical trials [8–11] and only one of the studies was a case series. A total of 346 subjects were included, 315 female and 25 male (366 joints). The follow-up period was between 168 days and 34.4 months. Most diagnoses were between Wilkes stage III and Wilkes stage IV. Before performing arthroscopic surgery, conservative treatment with NSAIDs, physical therapy, and an occlusal splint was attempted for 3 months [11, 12], or 6 months [8, 10]. All patients underwent arthroscopic surgery, but some additionally received injections of certain substances at the intra-articular level. Sodium hyaluronate was infiltrated into 179 joints [8, 9, 11, 12]. The PRGF was infiltrated into 92 joints [9, 10]. Regarding the improvement of pain and oral opening, the results and measurements were very heterogeneous. The viability of the dosage of the substances used and the different combinations did not allow further analysis. Subjects in all groups and with all substances reported improvement in jaw function and a reduction in pain.

The comparison between PRGF and 5% sodium chloride did not show statistically significant differences [9]. The comparison between PRGF and HA 1% shows that PRGF is more effective than HA in reducing pain [10]. Furthermore, when sodium hyaluronate was compared to Ringer’s lactate, the reduction in joint pain was statistically significant in the sodium hyaluronate group [8]. Finally, when lactated Ringer’s solution and HA were compared, a significant improvement in MIO was observed in both groups [12]. But in another study, HA compared to Ringer’s lactate alone found no benefit of HA as adjunctive therapy to arthroscopy over Ringer’s lactate alone [11] Table 2.

### Pooled Results from Interventions of Interest

#### VAS

**Fig. 2:**
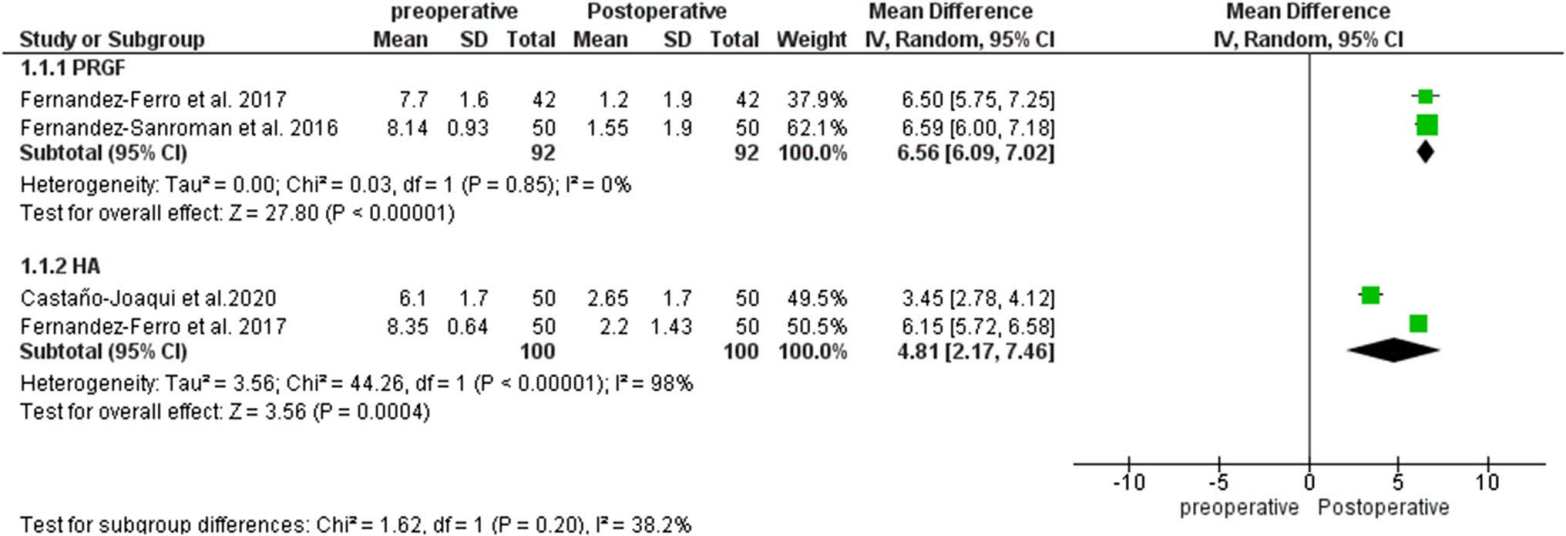
Forest plot for VAS between PRGF and HA

Pooled results for pain changes measured by VAS between 12 and 24 months for both the PRGF and HA groups showed favorable results for both therapies, MD: 6.56 95% CI (6.09–7.02) and MD 4.81 CI95% (2.17–7.46), respectively. The CI made it impossible to observe statistical differences between the two evaluated treatment subgroups (Fig. 2).

#### MMO

**Fig. 3:**
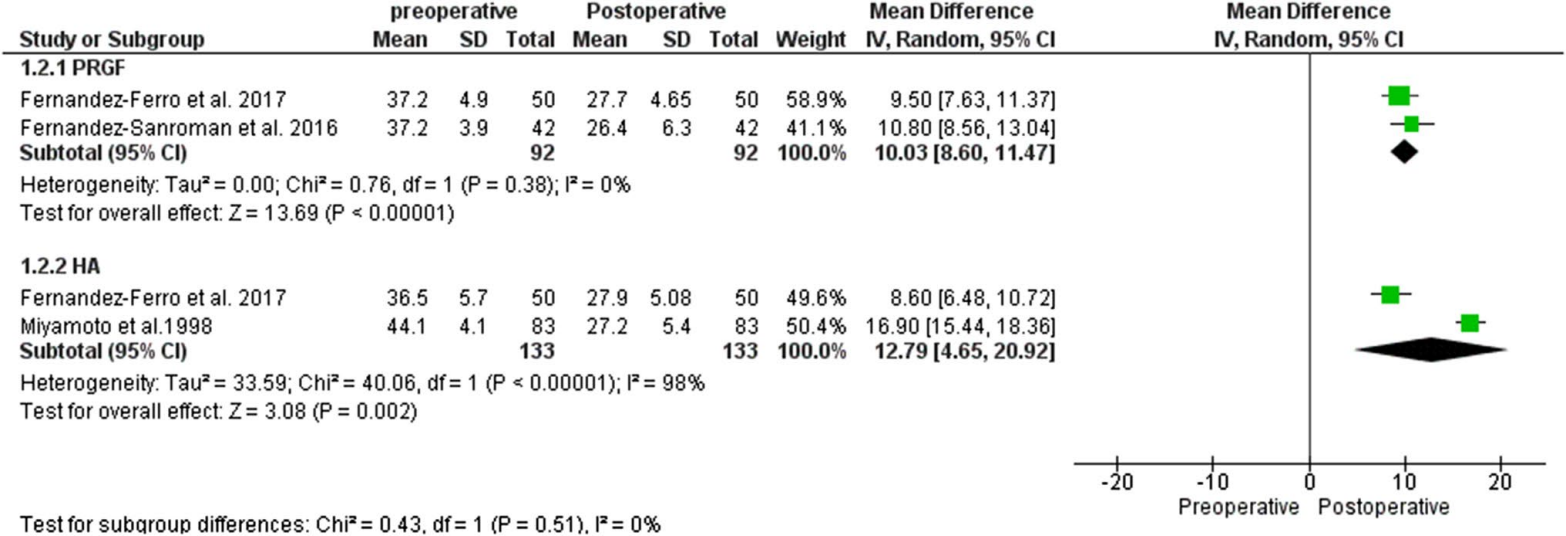
Forest plot for MMO between PRGF and HA

On the other hand, the comparison for MMO between pre- and postoperatively between 12 and 24 months for both the PRGF and HA groups also showed efficacy results for this outcome for the two treatments, MD: 10.03 95% CI (8.60–11.47) and MD 12.79 95% CI (4.65–20.92), respectively. The behavior of MMO between the two treatment subgroups was comparable (*p* = 0.51**)** (Fig. 3).

### Risk of Bias in Included Studies

**Fig. 4:**
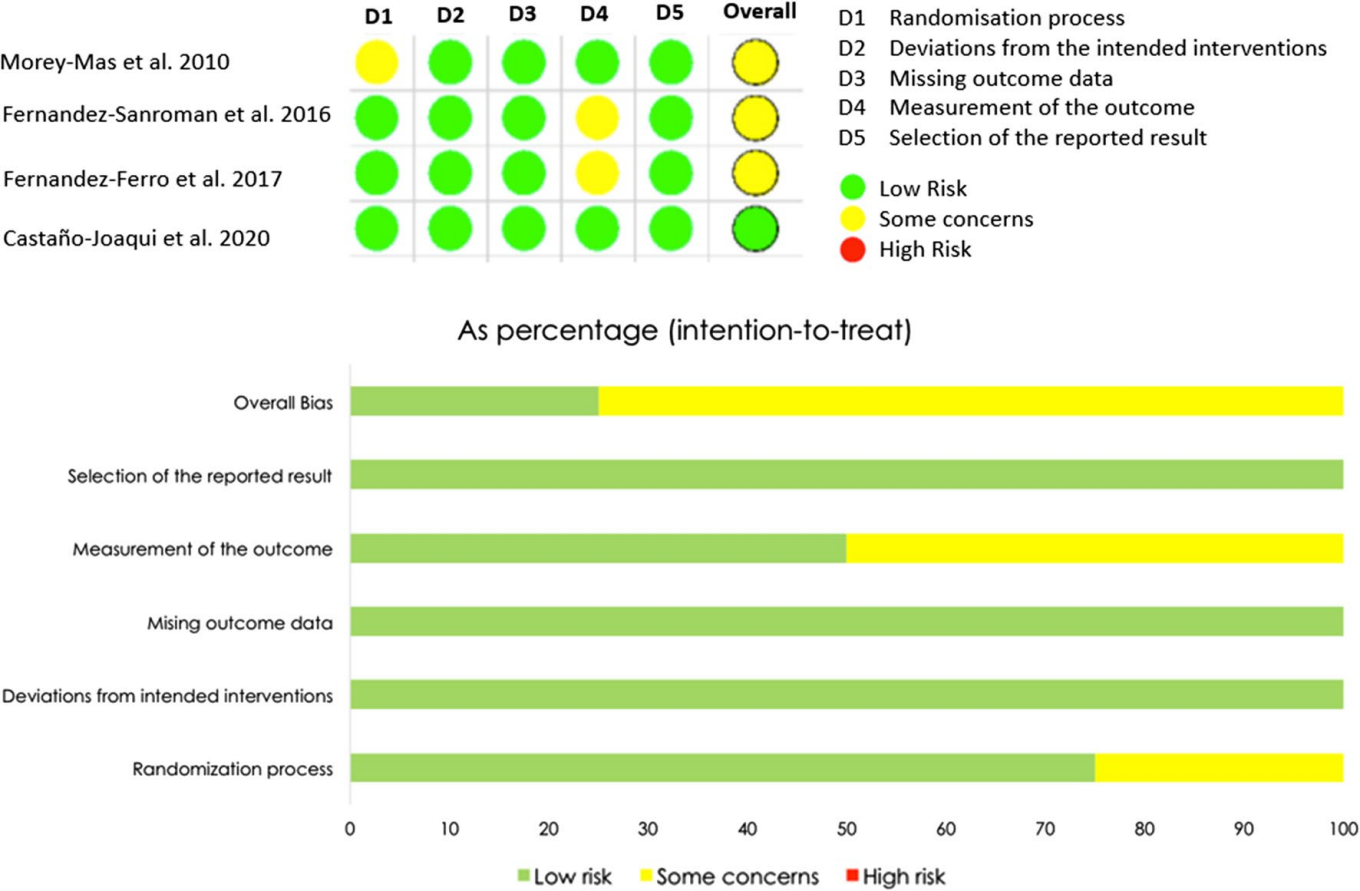
Risk of bias assesment

The article evaluated with the Joanna Briggs Institute Verification List (JBI) was classified as having an adequate, acceptable, or low-quality risk of bias since it only presented an unclear item [12]. Of the 4 randomized clinical trials included in the study and evaluated with the ROB2 tool, the majority showed results of “some concerns” [8–10] and only one study was considered “low risk” [11]. The most affected domain was domain 4 ‘Measurement of the outcome’ which was affected in two studies [9, 10]. Additionally, domain 1 ‘randomization process’ presented some concerns in one of the studies [8]. Finally, the most affected item was the question, ‘Were those delivering treatment blind to treatment assignment?’ due to the difficulty involved in hiding the substance to be injected intra-articularly from the surgeon who is going to perform the procedure. Nevertheless, in some of the articles, this bias was eliminated since the person who performed the postoperative measurements was blinded for study purposes Fig. 4.

## Discussion

Temporomandibular joint arthroscopy is an excellent tool for diagnosis and treatment, with adequate training. A review conducted in 2018 compared the results between operative arthroscopy and arthrocentesis, and variables such as maximum oral opening and pain were measured. Excellent results were obtained, ranging from 84 to 93% improvement for arthroscopy [13]. On the other hand, in 2019, arthroscopy was compared with open surgery in 78 joints, evaluating pain, noise, diet, quality of life, and disk position using magnetic resonance imaging, among other variables. The results were in favor of arthroscopic surgery, which presented greater and earlier improvement. The authors concluded that arthroscopic surgery shows an early improvement in clinical symptoms, and open surgery could be reserved for advanced cases [14]. Also, for the treatment of temporomandibular diseases, the injection of intra-articular substances has been a topic of great interest in the field of arthroscopy. In a systematic review by Sakalys et al. it was revealed that the injection of these substances has an important impact on the improvement of intra-articular pain, and it has been shown that these positive results are even greater with the use of plasma rich in growth factors compared to hyaluronic acid [15]. Another systematic review by Haigler et al. [16] revealed that the injection of substances such as plasma rich in growth factors, platelet-rich plasma, or hyaluronic acid could reduce pain but did not significantly increase mouth opening. However, they relate the low level of evidence to the data’s heterogeneity, suggesting more studies. Nevertheless, until the moment of our search, we did not find systematic reviews that analyze the different substances exclusively after arthroscopy, evaluating pain and maximum mouth opening (MMO).

### Hyaluronic Acid or Sodium Hyaluronate

Ferreira et al. published another systematic review of hyaluronic acid. These authors considered 21 articles that evaluated pain in patients with disk displacement and degenerative and osteoarthritic processes after injection of hyaluronic acid. However, it was not possible to adequately evaluate the results due to the heterogeneity of the evaluation and concentration dose, among others, which is why they suggest the development of established protocols to improve the evaluation of the results [17]. With regard to this substance, evidence of its use subsequent to arthroscopy can be found even since 1998, where Miyamoto et al. report a series of 83 joints that were subjected to this treatment with favorable results in relation to pain reduction and a significant improvement in maximum oral opening. The authors attribute these good results to the combination of arthroscopic techniques with hyaluronic acid injection that, in addition to releasing adhesions, allows the sweep of inflammatory mediators and improves the range of mandibular movements [12]. Additionally, what makes the results last over time is the capacity of sodium hyaluronate for its lubricating, protective, and repairing capacity in intra-articular tissue. On the other hand, it has an analgesic effect by blocking nerve endings on the intra-articular surface. This is consistent with the results found in studies that were compared with a control group where there was a significant improvement in pain and oral opening [8]. Contrary to these results, Castaño-Joaqui et al. found no benefit in terms of pain, maximal mouth opening (MMO), or even other measures. They suggest that the symptomatic improvement results may be due to arthroscopy, adjunctive drugs, and physiotherapy [11].

Concerning other substances, Fernandez-Ferro et al. compared HA with PRGF; however, although PRGF presented better results in improving pain and MMO, the differences were minimal. They attribute it to the characteristics of the injectable product for its potential in the treatment of degenerative processes and to the fact that the injection was carried out in both joint spaces, but they recommend further studies [10].

### Plasma-Rich Growth Factors

As previously mentioned, PRGF did not significantly improve compared to HA [10]. However, compared to a control group, PRGF seems to show better results in improving pain in the medium term between the first 6 to 12 postoperative months. Nevertheless, at 2 years of follow-up, it does not seem to have added significant improvements in pain or MMO compared to the group that did not receive the evaluated substance. Additionally, they found better magnetic resonance imaging findings with a significant decrease in joint effusion and osteoarthritic processes [9].

### Other Studies

A study carried out by González et al. in 2020 used platelet-rich fibrin in upper and lower articular space with good postoperative results in terms of oral opening postoperative pain based on the significance of growth factors that promote better and faster healing of intra-articular tissues. They also involve the inferior space using a minimally invasive technique. However, in this regard, there are no studies that study this substance with large patient samples or clinical studies that evaluate it with control groups (González et al. [18]).

Finally, within the methodological limitations of the current systematic review, it is found that due to the high heterogeneity between the different protocols of the interventions used in each one of the studies, it was only possible to combine the information for some treatments; however, we suggest that the information derived should be analyzed with caution since the comparison information is derived from different studies and therefore could be considered as an indirect comparison of the treatments. Consequently, observing trends derived before and after each subgroup was only possible.

## Conclusions

Multiple substances have been infiltrated into the temporomandibular joint, with sodium hyaluronate/hyaluronic being the most studied. However, variation in substance types, dosages, and frequencies makes assessment difficult. Therefore, the benefit of substances as adjuncts to TMJ arthroscopy has not been clearly established. Nevertheless, these results should be interpreted with caution due to the low number of cases and the limited description of their benefits.

## Appendix 1

Pubmed: (((“TMJ disorders” OR “TMJ disorder” OR “temporomandibular joint disorder” OR “temporomandibular disease” OR “temporomandibular diseases” OR “temporomandibular joint disorders” OR “temporomandibular disorder” OR “temporomandibular disorders” OR “temporomandibular joint disease” OR “temporomandibular joint diseases”) AND (arthroscopy OR “arthroscopic surgical procedure” OR “arthroscopic surgery”)) AND (“intraarticular injection” OR “intraarticular injections” OR “intra-articular injection” OR “intra-articular injections” OR “injections, intra-articular” OR “injection, intra-articular” OR “injection, intraarticular” OR “injections, intraarticular”)) AND (viscosupplementation OR “platelet-rich fibrin” OR “fibrin, platelet-rich” OR “platelet rich fibrin” OR “leukocyte and platelet-rich fibrin” OR analgesic OR analgesics OR “analgesic drug” OR “analgesic drugs” OR “analgesic agent” OR “analgesic agents” OR corticosteroid OR corticosteroids OR corticoid OR corticoids OR anesthetic OR anesthetics OR “anesthetic drugs” OR “anesthetic drug” OR “anesthetic agent” OR “anesthetic agents” OR “local anesthetic” OR “local anesthetics” OR “anesthetics, local” OR “anesthetic, local” OR “hyaluronic acid” OR “acid, hyaluronic” OR “sodium hyaluronate” OR “hyaluronate sodium” OR “platelet-rich plasma” OR “platelet rich plasma” OR “plasma, platelet-rich”).

Embase: (‘tmj disorders’ OR ‘tmj disorder’ OR ‘temporomandibular joint disorder’ OR ‘temporomandibular joint disorders’/exp OR ‘temporomandibular joint disorders’ OR ‘temporomandibular disorder’/exp OR ‘temporomandibular disorder’ OR ‘temporomandibular disorders’ OR ‘temporomandibular joint disease’/exp OR ‘temporomandibular joint disease’ OR ‘temporomandibular joint diseases’/exp OR ‘temporomandibular joint diseases’ OR ‘temporomandibular joint’ OR ‘joint, temporomandibular’ OR tmj) AND (arthroscopy OR ‘arthroscopic surgical procedure’ OR ‘arthroscopic surgery’) AND (‘intraarticular injection’ OR ‘intraarticular injections’ OR ‘intra-articular injection’ OR ‘intra-articular injections’ OR ‘injections, intra-articular’ OR ‘injection, intra-articular’ OR ‘injection, intraarticular’ OR ‘injections, intraarticular’ OR injection OR injections OR viscosupplementation OR ‘platelet-rich fibrin’ OR ‘fibrin, platelet-rich’ OR ‘platelet rich fibrin’ OR ‘leukocyte and platelet-rich fibrin’ OR analgesic OR analgesics OR ‘analgesic drug’ OR ‘analgesic drugs’ OR ‘analgesic agent’ OR ‘analgesic agents’ OR corticosteroid OR corticosteroids OR corticoid OR corticoids OR anesthetic OR anesthetics OR ‘anesthetic drugs’ OR ‘anesthetic drug’ OR ‘anesthetic agent’ OR ‘anesthetic agents’ OR ‘local anesthetic’ OR ‘local anesthetics’ OR ‘anesthetics, local’ OR ‘anesthetic, local’ OR ‘hyaluronic acid’ OR ‘acid, hyaluronic’ OR ‘sodium hyaluronate’ OR ‘hyaluronate sodium’ OR ‘platelet-rich plasma’ OR ‘platelet rich plasma’ OR ‘plasma, platelet-rich’).

Scopus: TITLE ABS-KEY (“TMJ disorders” OR “TMU disorder” OR “temporomandibular joint disorder” OR “temporomandibular joint disorders” OR “temporomandibular disorder” OR “temporomandibular disorders” OR “temporomandibular joint disease” OR “temporomandibular joint diseases” OR “temporomandibular joint” OR “Joint, Temporomandibular” OR tmj)) AND (TITLE-ABS-KEY (arthroscopy OR “arthroscopic surgical procedure” OR “arthroscopic surgery”)) AND (TITLE-ABS-KEY (“intraarticular injection” OR “intraarticular injections” OR “intra-articular injection” OR “intra-articular injections” OR injection OR injections OR viscosupplementation OR “platelet-rich fibrin” OR “platelet rich fibrin” OR “leukocyte and platelet-rich fibrin” OR analgesic OR analgesics OR “analgesic drug” OR “analgesic drugs” OR “analgesic agent” OR “analgesic agents” OR corticosteroid OR corticosteroids OR corticoid OR corticoids OR anesthetic OR anesthetics OR “anesthetic drugs” OR “anesthetic drug” OR “anesthetic agent” OR “anesthetic agents” OR “local anesthetic” OR “local anesthetics” OR “hyaluronic acid” OR “sodium hyaluronate” OR “hyaluronate sodium” OR “platelet-rich plasma” OR “platelet rich plasma”)).

## Declarations

### Guide for Review Process

-Page MJ, McKenzie JE, Bossuyt PM, Boutron I, Hoffmann TC, Mulrow CD, et al. The PRISMA 2020 statement: an updated guideline for reporting systematic reviews. BMJ 2021;372:n71.

-Sterne JAC, Savović J, Page MJ, Elbers RG, Blencowe NS, et al.: RoB 2: a revised tool for assessing risk of bias in randomised trials. BMJ 366: l4898, 2019.

## References

[1] Silva MAG, Pantoja LLQ, Dutra-Horstmann KL (2020). Prevalence of degenerative disease in temporomandibular disorder patients with disk displacement: A systematic review and meta-analysis. J Craniomaxillofac Surg.

[2] Al-Moraissi EA, Wolford LM, Ellis E (2020). The hierarchy of different treatments for arthrogenous temporomandibular disorders: A network meta-analysis of randomized clinical trials. J Craniomaxillofac Surg.

[3] Ohnishi M (1975). Arthroscopy of the temporomandibular joint. J Jpn Stomat.

[4] McCain JP, Podrasky AE, Zabiegalski NA (1992). Arthroscopic disk repositioning and suturing: a preliminary report. J Oral Maxillofac Surg.

[5] Martín-Granizo R, Millón-Cruz A (2016). diskopexy using resorbable pins in temporomandibular joint arthroscopy: clinical and magnetic resonance imaging medium-term results. J Craniomaxillofac Surg.

[6] Millón-Cruz A, Martín-Granizo López R (2020). Long-term clinical outcomes of arthroscopic diskopexy with resorbable pins. J Craniomaxillofac Surg.

[7] Loureiro Sato FR, Tralli G (2020). Arthroscopic diskopexy technique with anchors for treatment of temporomandibular joint internal derangement: clinical and magnetic resonance imaging evaluation. J Craniomaxillofac Surg.

[8] Morey-Mas MA, Caubet-Biayna J, Varela-Sende L (2010). Sodium hyaluronate improves outcomes after arthroscopic lysis and lavage in patients with Wilkes stage III and IV disease. J Oral Maxillofac Surg.

[9] Fernández Sanromán J, Fernández Ferro M, Costas López A (2016). Does injection of plasma rich in growth factors after temporomandibular joint arthroscopy improve outcomes in patients with Wilkes stage IV internal derangement? A randomized prospective clinical study. Int J Oral Maxillofac Surg.

[10] Fernández-Ferro M, Fernández-Sanromán J, Blanco-Carrión A, Costas-López A, López-Betancourt A, Arenaz-Bua J, Stavaru Marinescu B (2017). Comparison of intra-articular injection of plasma rich in growth factors versus hyaluronic acid following arthroscopy in the treatment of temporomandibular dysfunction: a randomised prospective study. J Craniomaxillofac Surg.

[11] Castaño-Joaqui OG, Cano-Sánchez J, Campo-Trapero J (2021). TMJ arthroscopy with hyaluronic acid: a 12-month randomized clinical trial. Oral Dis.

[12] Miyamoto H, Sakashita H, Miyata M (1998). Arthroscopic management of temporomandibular closed lock. Aust Dent J.

[13] Laskin DM (2018). Arthroscopy versus arthrocentesis for treating internal derangements of the temporomandibular joint. Oral Maxillofac Surg Clin North Am.

[14] Abdelrehem A, Kai HY, Zheng JS (2019). Arthroscopic versus open disk repositioning in management of temporomandibular joint internal derangement. Int J Oral Maxillofac Surg.

[15] Sakalys D, Dvylys D, Simuntis R (2020). Comparison of different intraarticular injection substances followed by temporomandibular joint arthroscopy. J Craniofac Surg.

[16] Haigler MC, Abdulrehman E, Siddappa S (2018). Use of platelet-rich plasma, platelet-rich growth factor with arthrocentesis or arthroscopy to treat temporomandibular joint osteoarthritis: Systematic review with meta-analyzes. J Am Dent Assoc.

[17] Ferreira N, Masterson D, de Lima RL (2018). Efficacy of viscosupplementation with hyaluronic acid in temporomandibular disorders: A systematic review. J Craniomaxillofac Surg.

[18] González LV, López JP, Díaz-Báez D (2021). Clinical outcomes of operative arthroscopy and temporomandibular medical infiltration with platelet-rich fibrin in upper and lower articular space. J Craniomaxillofac Surg.

